# Exosomal MALAT1 from Rapid Electrical Stimulation-Treated Atrial Fibroblasts Activates Autophagy by Downregulating miR-204-5p and Upregulating LC3B

**DOI:** 10.3390/cells15121126

**Published:** 2026-06-22

**Authors:** Su-Kiat Chua, Bao-Wei Wang, Ying-Ju Yu, Wei-Jen Fang, Chiu-Mei Lin, Cheng-Yen Chuang, Kou-Gi Shyu

**Affiliations:** 1 School of Medicine, College of Medicine, Fu Jen Catholic University, New Taipei 24205, Taiwan; mei882153@gmail.com; 2 Division of Cardiology, Department of Internal Medicine, Shin Kong Wu Ho-Su Memorial Hospital, Taipei 11101, Taiwan; baowei@ms22.hinet.net (B.-W.W.); kinki1983yu@gmail.com (Y.-J.Y.); wjfang0719@gmail.com (W.-J.F.); medicine.chuang@gmail.com (C.-Y.C.); shyukg@ms12.hinet.net (K.-G.S.); 3 Department of Emergency Medicine, Shin Kong Wu Ho-Su Memorial Hospital, Taipei 11101, Taiwan

**Keywords:** atrial fibrillation, MALAT1, miR-204-5p, LC3B, autophagy, rapid electrical stimulation, exosome, competing endogenous RNA

## Abstract

**Highlights:**

**What are the main findings?**

- RES selectively induces exosomal MALAT1 secretion in human atrial fibroblasts (HCF-aa), which acts as a competing endogenous RNA (ceRNA) to putatively sequester miR-204-5p, thereby de-repressing LC3B expression and activating autophagy.
- Luciferase reporter assays, gain- and loss-of-function experiments, and immunofluorescence confocal microscopy confirm a MALAT1/miR-204-5p/LC3B signaling axis as a mechanistic driver of autophagic activation in atrial fibroblasts under rapid electrical stress.

**What are the implications of the main findings?**

- Exosomal MALAT1 extends its role as a master orchestrator of non-coding RNA networks in atrial fibroblasts, adding regulation of autophagy to its previously established functions in apoptosis (miR-499a-5p/SOX6) and electrical remodeling (miR-1/Tbx18/Cx43).
- The MALAT1/miR-204-5p/LC3B axis represents a potential therapeutic target for modulating autophagy-mediated atrial structural remodeling in atrial fibrillation.

**Abstract:**

**Background:** Atrial fibrillation (AF) is the most common sustained cardiac arrhythmia and is strongly associated with atrial structural remodeling driven by activated cardiac fibroblasts. Autophagy has been implicated in AF-related atrial remodeling; however, the non-coding RNA mechanisms that govern autophagic activation in atrial fibroblasts under rapid electrical stress remain poorly understood. **Methods:** Human cardiac fibroblasts from adult atria (HCF-aa) were subjected to rapid electrical stimulation (RES) at 0.5 V/cm and 10 Hz. Expression levels of exosomal metastasis-associated lung adenocarcinoma transcript 1 (MALAT1), cytoplasmic miR-204-5p, and microtubule-associated protein light chain 3B (LC3B) were measured using quantitative real-time PCR and Western blot analyses. Luciferase reporter assays were performed to confirm direct molecular interactions. The functional roles of MALAT1 siRNA, miR-204-5p mimics/antagomirs, rapamycin, and 3-methyladenine (3-MA) on LC3B expression and autophagic activation were assessed by Western blot and immunofluorescence confocal microscopy for LC3B puncta formation. **Results:** RES significantly induced exosomal MALAT1 expression in a voltage- and time-dependent manner, peaking at 2 h post-stimulation, while cytoplasmic MALAT1 levels remained unchanged. Cytoplasmic miR-204-5p exhibited an initial transient rise followed by a significant decline at 2 h, inversely correlating with peak MALAT1 levels. LC3B mRNA and protein expression subsequently increased, peaking at 6 and 16 h, respectively. Luciferase reporter assays confirmed that miR-204-5p directly binds both the MALAT1 transcript and the 3′-UTR of LC3B mRNA. MALAT1 knockdown augmented miR-204-5p levels and suppressed LC3B expression, while miR-204-5p overexpression attenuated RES-induced LC3B upregulation and LC3B puncta accumulation. Conversely, miR-204-5p inhibition further enhanced autophagic activation, as evidenced by increased LC3B puncta density. **Conclusions:** In HCF-aa subjected to RES, MALAT1 functions intracellularly as a competing endogenous RNA to putatively sequester miR-204-5p, thereby de-repressing LC3B expression and promoting autophagic activation. Concurrent exosomal secretion of MALAT1 may additionally serve as a paracrine signal to neighboring cells, though this requires future conditioned-media transfer experiments to confirm.

## 1. Introduction

Atrial fibrillation (AF) is the most common sustained cardiac arrhythmia in clinical practice, affecting over 33 million individuals worldwide and carrying a substantially elevated risk of stroke, heart failure, and overall mortality [1]. Tachycardia-induced atrial structural and electrical remodeling are two cardinal features of AF pathogenesis [2]. Cardiac fibroblasts, long regarded as passive structural sentinels, are now recognized as active participants in atrial remodeling through fibroblast–myocyte coupling, whereby electrically silent fibroblasts modulate cardiomyocyte action potentials, thereby perpetuating the arrhythmogenic substrate [3]. Rapid electrical stimulation (RES) of human cardiac fibroblasts from adult atria (HCF-aa) has been established as a validated in vitro model that faithfully recapitulates key features of AF-associated fibroblast activation [4,5].

Recent studies have highlighted the pivotal role of long non-coding RNAs (lncRNAs) and microRNAs (miRNAs) in the molecular regulation of AF [6]. Metastasis-associated lung adenocarcinoma transcript 1 (MALAT1) is a highly conserved lncRNA that modulates gene expression as a competing endogenous RNA (ceRNA), sequestering specific miRNAs and thereby derepressing their target messenger RNAs [7]. Exosomes—nanoscale extracellular vesicles that carry diverse non-coding RNA cargo and mediate intercellular communication—play a critical role in transmitting MALAT1-mediated signals in cardiovascular biology [8]. We have previously demonstrated that RES of HCF-aa significantly induces exosomal MALAT1 secretion, and that this exosomal MALAT1 sponges miR-499a-5p to upregulate SOX6 expression and modulate fibroblast apoptosis [4]. In a related study, we further showed that exosomal miR-1 released by RES-exposed HCF-aa represses Tbx18 and consequently upregulates connexin 43 (Cx43) expression [5]. These findings suggest that exosomal MALAT1 orchestrates a broader non-coding RNA network in electrically stressed atrial fibroblasts; however, additional downstream targets of this axis remain to be identified.

MicroRNA-204-5p (miR-204-5p) has emerged as a clinically relevant miRNA in AF and is significantly downregulated in the coronary sinus plasma of AF patients [9]. Mechanistically, miR-204-5p functions as a potent suppressor of autophagy by directly targeting microtubule-associated protein light chain 3B (LC3B): seminal work demonstrated that miR-204 binds the 3′-UTR of LC3B to inhibit autophagic activation, a finding subsequently validated in cardiac ischemia–reperfusion models [10,11]. The conversion of cytosolic LC3B-I to its lipidated autophagosome form, LC3B-II, is a defining hallmark of autophagy activation and has been observed to be dysregulated in both human atrial tissues from AF patients and in animal models of rapid atrial pacing, implicating autophagy as a potential contributor to atrial electrical remodeling [12,13]. Despite these observations, whether MALAT1 regulates miR-204-5p and thereby modulates LC3B-mediated autophagy in atrial fibroblasts under conditions of rapid electrical stress has not been investigated.

The present study aims to elucidate a novel regulatory axis involving exosomal MALAT1, miR-204-5p, and LC3B in HCF-aa subjected to RES. We hypothesize that RES-induced exosomal MALAT1 putatively sequesters miR-204-5p, thereby derepressing LC3B expression and activating autophagy in atrial fibroblasts. Elucidating this signaling cascade may advance our understanding of AF pathogenesis and identify potential therapeutic targets for atrial fibroblast-mediated remodeling.

## 2. Materials and Methods

### 2.1. Cell Culture of HCF-aa

Human cardiac fibroblasts-adult atrial (HCF-aa) were obtained from ScienCell Research Laboratories (San Diego, CA, USA). These cells were cultured in fibroblast medium supplemented with essential and nonessential amino acids, vitamins, hormones, growth factors, and 2% fetal bovine serum. The medium was buffered with HEPES and bicarbonate to maintain a stable pH of 7.4. The cells were incubated in a humidified 5% CO_2_ atmosphere at 37 °C to ensure optimal growth. For all experimental protocols, HCF-aa were used at passages 3 to 6 to maintain phenotypic consistency.

### 2.2. In Vitro Rapid Electrical Stimulation (RES)

The HCF-aa were subjected to RES using an established protocol. Upon reaching confluence, the culture medium was replaced with serum-free Fibroblast Medium (ScienCell, Cat No: 2301, Carlsbad, CA, USA) for 6 h prior to stimulation. Cells were then transferred to an EPS-01 culture pacer dish (BioEast, Taipei, Taiwan) equipped with parallel platinum electrodes to prevent electrolysis. The RES was delivered as a unipolar square waveform at 10 Hz (duration, 50 ms; interval, 50 ms). A voltage gradient of 0.5 V/cm was applied. Control cells were maintained under identical conditions without electrical stimulation.

### 2.3. Extraction of Exosomes from Cell Media

Exosomes were isolated from cell culture media using the Total Exosome Isolation Reagent (Invitrogen, Thermo Fisher Scientific, Waltham, MA, USA) according to the manufacturer’s guidelines. Briefly, the cell-free media were centrifuged at 2000× *g* for 30 min to remove cellular debris. The resulting supernatant was mixed with the isolation reagent at a 2:1 ratio and incubated overnight at 4 °C. The mixture was then centrifuged at 10,000× *g* for 1 h at 4 °C. The exosome pellet was resuspended in phosphate-buffered saline (PBS) and quantified using an ExoQuant™ quantification assay kit (BioVision, Milpitas, CA, USA).

Using this protocol, isolated vesicles have previously been confirmed to fall within the expected exosomal size range of 50–150 nm, and exosomal identity has been validated by CD9 and CD81 Western blots in our prior publications employing the same isolation platform. Comprehensive characterization per MISEV2023 guidelines, including nanoparticle tracking analysis, transmission electron microscopy, and full tetraspanin marker profiling, was not performed in the present study and is acknowledged as a limitation [14].

### 2.4. Reverse Transcription and Real-Time Quantitative PCR

Total cytoplasmic RNA was extracted using TRIzol reagent (Invitrogen, Thermo Fisher Scientific, Waltham, MA, USA) according to the manufacturer’s protocol. For exosomal RNA extraction, the Total Exosome RNA and Protein Isolation Kit (Invitrogen, Thermo Fisher Scientific) was used as previously described. Prior to RNA extraction, exosome quantity was standardized across all experimental conditions using the ExoQuant™ quantification assay kit (BioVision, Milpitas, CA, USA) to ensure equal exosome input. RNA quality of each exosome preparation was confirmed using a BiOptic Qsep100 analyzer (BiOptic Inc., Taipei, Taiwan) with an RNA cartridge kit; all samples were analyzed in duplicate, and electropherogram profiles were generated to verify RNA integrity before proceeding to reverse transcription.

Reverse transcription of total RNA was performed using random hexamers and a High-Capacity cDNA Reverse Transcription Kit (Applied Biosystems, Thermo Fisher Scientific, Waltham, MA, USA). Quantitative PCR was conducted using Fast SYBR^®^ Green Master Mix (Applied Biosystems, Thermo Fisher Scientific, Waltham, MA, USA) on an ABI StepOnePlus cycler with the following cycling conditions: 95 °C for 15 min, followed by 40 cycles of 94 °C for 15 s, 55 °C for 30 s, and 70 °C for 30 s.

Relative gene expression levels were calculated using the 2^−ΔCT^ method, where ΔCT = CT(target gene) − CT(control condition), as previously described. All expression values are presented as fold-change relative to the unstimulated control group, which was assigned a reference value of 1.0. This method normalizes expression to the matched experimental control condition and does not require a housekeeping gene, consistent with the approach used in our prior peer-reviewed work employing the same experimental platform.

### 2.5. Construction of Expression Vectors and Transfection

To confirm the direct interaction between miR-204-5p and MALAT1, a 500 bp human MALAT1 DNA fragment encompassing the predicted miR-204-5p binding site (nucleotides 1650–1671; binding site sequence: 5′-AAGAGUAGCAUGAGGAAGGAAA-3′) was artificially synthesized and cloned into a pUC57 intermediate vector. The insert was subsequently digested with restriction enzymes and ligated into the pmirNanoGLO luciferase reporter vector (Promega, Madison, WI, USA) to generate the wild-type MALAT1 reporter construct. Similarly, a 500 bp human LC3B 3′-UTR fragment encompassing the predicted miR-204-5p binding site (nucleotides 41–62; binding site sequence: 5′-UUACCAAGGAAAAAAAAGGGAU-3′) was cloned using the same strategy. Mutant constructs were generated by site-directed mutagenesis of the conserved miR-204-5p binding sites within each 500 bp fragment. All cloned plasmids were confirmed by DNA sequencing. This approach was performed as previously described.

Cells were transfected at 60–75% confluence using ViaFect™ Transfection Reagent (Promega) or Lipofectamine RNAiMax (Thermo Fisher Scientific, Waltham, MA, USA) according to the manufacturer’s protocols. The miR-204-5p mimics (5′-UUCCCUUUGUCAUCCUAUGCCU-3′), antagomirs (5′-AGGCAUAGGAUGACAAAGGGAA-3′), and MALAT1 siRNA (Cat. no. 4392420, Thermo Fisher Scientific) were delivered by transfection as specified for each experimental condition.

### 2.6. Luciferase Activity Assay

#### 2.6.1. 3′-UTR Luciferase Binding Assay

To confirm direct miR-204-5p binding to MALAT1 and the LC3B 3′-UTR, the 500 bp wild-type and mutant reporter constructs described in Section 2.5 were cloned into the pmirNanoGLO dual luciferase reporter vector (Promega, Madison, WI, USA), as previously described. HCF-aa were co-transfected with reporter plasmids and miR-204-5p wild-type or mutant mimics using ViaFect™ Transfection Reagent (Promega). After 24 h, luciferase activity was measured using the Nano-Glo Dual-Luciferase Reporter Assay System (Promega) on a Glomax Multi Detection System luminometer (Promega). Firefly luciferase activity was normalized to Renilla luciferase activity to control for transfection efficiency. This assay evaluates miRNA-mediated post-transcriptional repression via direct 3′-UTR binding, rather than transcriptional activity.

#### 2.6.2. Promoter Activity Assay

To evaluate transcriptional regulation, human promoter constructs were generated and ligated into the pGL3-basic luciferase plasmid vector (Mission Biotech, Taipei, Taiwan). Plasmids were transfected into HCF-aa using ViaFect™ Transfection Reagent (Promega). Luciferase activity was measured as previously described to determine promoter-driven transcriptional activity in response to RES and molecular interventions. This assay is entirely independent of the 3′-UTR binding system described in Section 2.6.1.

### 2.7. Western Blot Analysis

Cells were harvested by scraping and centrifuged at 300× *g* for 10 min at 4 °C. The resulting pellet was resuspended, homogenized in RIPA Lysis and Extraction Buffer (Thermo Fisher Scientific, Rockford, IL, USA), and centrifuged at 14,000× *g* for 15 min at 4 °C. Protein content was determined using a Bio-Rad Protein Assay (Bio-Rad Laboratories, Hercules, CA, USA). Equal amounts of protein (30 μg per lane) were loaded onto 15% sodium dodecyl sulfate-polyacrylamide gels for optimal resolution of LC3B-I (~16 kDa) and LC3B-II (~14 kDa), and subjected to electrophoresis. Proteins were transferred to polyvinylidene difluoride (PVDF) membranes (0.2 μm pore size; Merck Millipore, Darmstadt, Germany) by wet transfer at 100 V for 90 min at 4 °C. Membranes were blocked with 5% non-fat dry milk in Tris-buffered saline with 0.1% Tween-20 (TBST) for 1 h at room temperature, followed by overnight incubation at 4 °C with primary antibodies against LC3B (Santa Cruz Biotechnology, Cat. no. sc-271837, Dallas, TX, USA) and β-actin (mouse monoclonal anti-β-actin, Sigma-Aldrich, Saint Louis, MO, USA). After washing with TBST, membranes were incubated with horseradish peroxidase-conjugated secondary antibodies for 1 h at room temperature, and signals were detected using enhanced chemiluminescence (ECL) reagent (Thermo Fisher Scientific). To ensure linearity of detection for LC3B-I/II quantification, all samples were confirmed to fall within the linear range of exposure prior to densitometric analysis. Band densitometry was performed using ImageJ software (version 1.54; National Institutes of Health, Bethesda, MD, USA). Equal protein loading was further verified by quantifying β-actin band intensity. The LC3B-II/LC3B-I ratio, normalized to β-actin, was used as the primary metric for quantifying autophagy. The detailed procedure has been described previously [4,15].

### 2.8. Immunofluorescence Staining

HCF-aa were fixed in 4% paraformaldehyde for 1 h and washed with PBS. The cells were incubated with a primary antibody against LC3B at 4 °C for 12 h, followed by a fluorescently conjugated secondary antibody for 1–2 h in the dark. Nuclei were stained with DAPI, and the cytoskeleton was visualized with phalloidin. Samples were examined using confocal microscopy, and at least three random fields were captured per sample to quantify LC3B puncta density.

### 2.9. Statistical Analysis

Data are presented as mean ± standard deviation (SD) from at least three independent experiments. Statistical significance was assessed using one-way analysis of variance (ANOVA), followed by Tukey–Kramer post hoc tests for multiple comparisons. A *p*-value of less than 0.05 was considered statistically significant. One-way ANOVA was applied under the assumption of approximate normality, which is biologically justified for fold-change expression data derived from independent controlled experiments, and is consistent with our previously published methodology [4,15]. We acknowledge that formal normality testing is not feasible with *n* = 3; non-parametric validation using the Kruskal–Wallis test with larger sample sizes is recommended for future studies.

## 3. Results

### 3.1. RES Up-Regulates Exosomal MALAT1 Expression in a Dose- and Time-Dependent Manner

**Figure 1:**
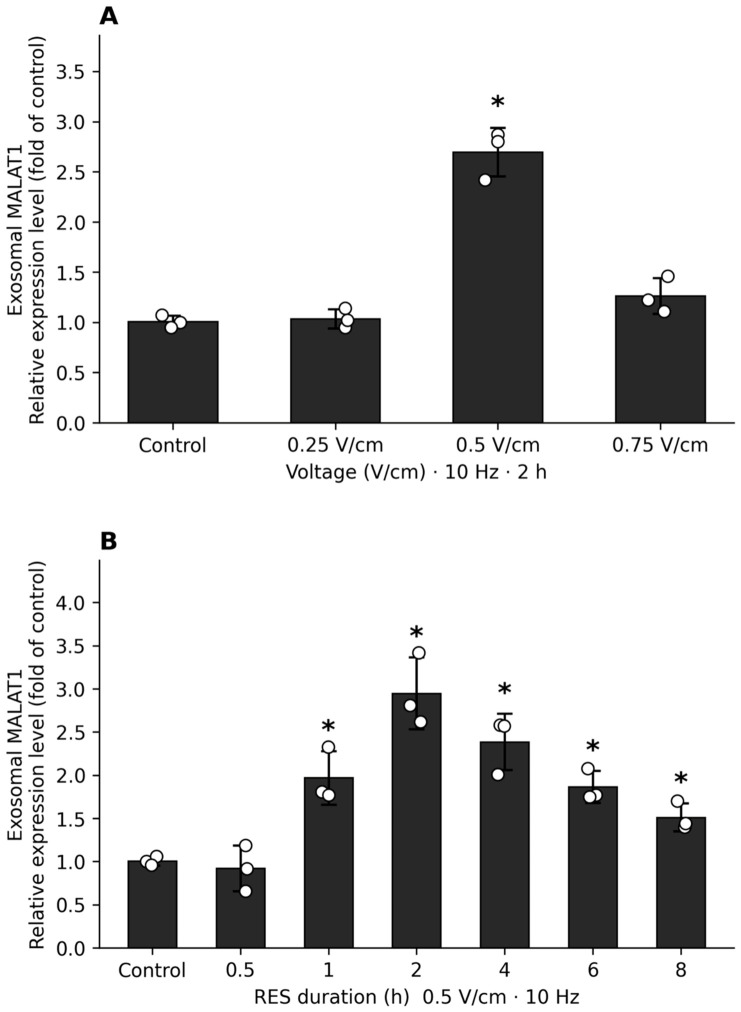
Rapid electrical stimulation (RES) induces the expression of exosomal MALAT1 in HCF-aa. (**A**) Exosomal MALAT1 mRNA expression levels in HCF-aa subjected to RES at varying voltage gradients (0.25, 0.5, and 0.75 V/cm) at 10 Hz for 2 h. (**B**) Time-dependent changes in exosomal MALAT1 mRNA levels in HCF-aa exposed to RES (0.5 V/cm and 10 Hz) for 0.5 to 8 h. The control group was cultured for 8 h without RES. Relative MALAT1 expression was determined by qPCR and normalized to the control group. The open circles are the individual data points. Data represent mean ± SD (*n* = 3 per group). * *p* < 0.001 versus the control group.

To investigate whether RES modulates MALAT1 secretion via exosomes, we performed dose–response and time-course experiments in HCF-aa. As illustrated in Figure 1A, exposure to RES at 0.5 V/cm and 10 Hz for 2 h resulted in a significant induction of exosomal MALAT1 expression (2.7 ± 2.4-fold relative to control, *p* < 0.001). In contrast, lower (0.25 V/cm; 1.04 ± 0.09-fold) and higher (0.75 V/cm; 1.26 ± 0.18-fold) voltage gradients did not significantly increase MALAT1 levels compared with the control group (1.01 ± 0.06-fold). Based on these findings, a voltage intensity of 0.5 V/cm was selected for all subsequent investigations.

The temporal dynamics of this induction followed a distinct bell-shaped pattern (Figure 1B). While no significant change was observed at 0.5 h (0.92 ± 0.27-fold), exosomal MALAT1 levels rose significantly at 1 h (1.97 ± 0.31-fold, *p* < 0.001) and reached a peak at 2 h post-stimulation (2.95 ± 0.42-fold, *p* < 0.001). Following the peak, MALAT1 levels gradually declined but remained significantly elevated through 8 h (1.51 ± 0.17-fold) compared to the unstimulated control group (1.01 ± 0.05-fold).

### 3.2. Reciprocal Expression of miR-204-5p and LC3B in Response to RES

**Figure 2:**
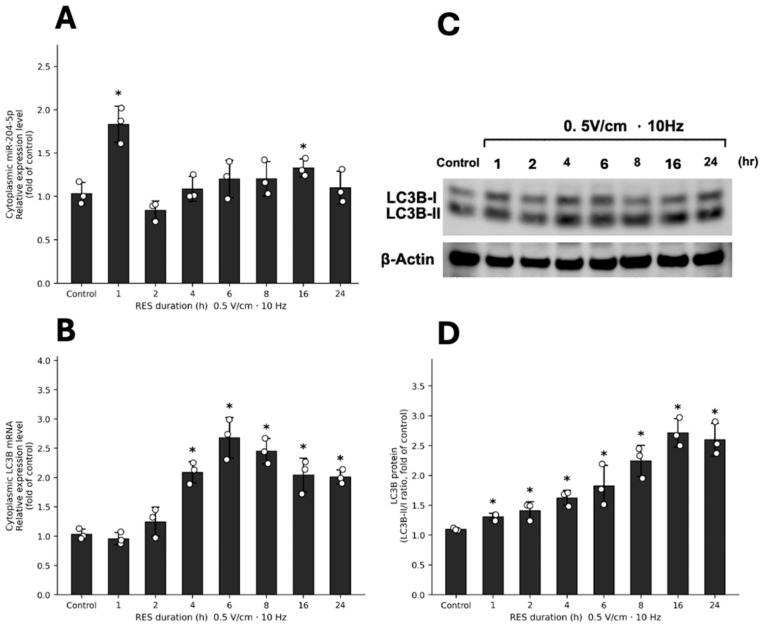
Rapid electrical stimulation (RES) modulates miR-204-5p, LC3B mRNA, and LC3B protein expression in HCF-aa. (**A**) Time-course analysis of cytoplasmic miR-204-5p expression levels in HCF-aa subjected to RES (0.5 V/cm, 10 Hz) for 1 to 24 h. (**B**) Quantitative expression of cytoplasmic LC3B mRNA in HCF-aa under the same RES conditions. (**C**) Representative Western blot images showing LC3B-I, LC3B-II, and β-actin in HCF-aa subjected to RES for 1 to 24 h; original gel images are provided in Supplementary Figure S1. (**D**) Quantitative analysis of LC3B protein expression (LC3B-II/I ratio) normalized to β-actin. The open circles are the individual data points. The control group was cultured for 24 h without RES. Data represent mean ± SD (*n* = 3 per group). * *p* < 0.05 versus the control group.

As shown in Figure 2A, cytoplasmic miR-204-5p expression exhibited a transient increase at 1-h post-RES (1.83 ± 0.21-fold, *p* < 0.05), followed by a significant decline at 2 h (0.84 ± 0.11-fold, *p* < 0.05 vs. 1 h), falling below the control group’s baseline (1.03 ± 0.13). This reduction in miR-204-5p expression coincided precisely with the peak levels of exosomal MALAT1 observed in Figure 1, supporting the hypothesis that MALAT1 acts as a ceRNA to putatively sequester miR-204-5p.

In parallel, we observed a delayed but robust induction of LC3B mRNA expression (Figure 2B). Following the nadir of miR-204-5p at 2 h, LC3B mRNA levels began to rise significantly at 4 h (2.09 ± 0.18-fold, *p* < 0.05 vs. control) and reached a peak at 6 h (2.68 ± 0.34-fold, *p* < 0.05 vs. control). LC3B expression remained significantly elevated (approximately 2.0-fold) through 24 h of continuous RES.

To confirm whether the RES-induced increase in LC3B mRNA translates to functional protein expression, we performed Western blot analysis to detect LC3B-I and LC3B-II levels in HCF-aa (original gel images are provided in Supplementary Figure S1; Figure 2C). Consistent with our mRNA findings, RES elicited a robust, time-dependent accumulation of LC3B protein (Figure 2D). While early stages of RES (1–2 h) showed only modest increases (1.31 ± 0.06 and 1.41 ± 0.15-fold, respectively), a significant induction was observed starting at 4 h (1.75 ± 0.32-fold, *p* < 0.05 vs. control). Protein expression continued to rise, reaching a peak at 16 h (2.71 ± 0.24-fold) and maintaining a high plateau through 24 h (2.60 ± 0.27-fold).

### 3.3. MALAT1 Regulates LC3B Expression by Sponging miR-204-5p

**Figure 3:**
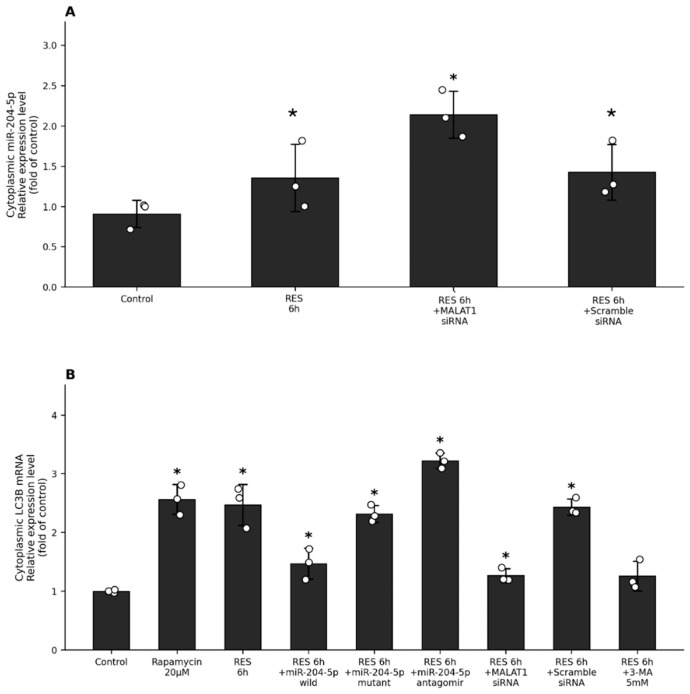
MALAT1 acts as a ceRNA for miR-204-5p to regulate LC3B expression in HCF-aa under RES. (**A**) Cytoplasmic miR-204-5p expression levels in HCF-aa subjected to RES (0.5 V/cm, 10 Hz for 6 h) with or without MALAT1 siRNA or scramble siRNA transfection. (**B**) Relative LC3B mRNA expression levels in HCF-aa under various experimental conditions, including treatment with rapamycin (20 μM), miR-204-5p wild-type/mutant mimics, miR-204-5p antagomir, MALAT1 siRNA, and 3-methyladenine (3-MA, 5 mM). The open circles are the individual data points. Data represent mean ± SD (*n* = 3). * *p* < 0.05 versus the control group.

As shown in Figure 3A, while RES (0.5 V/cm, 10 Hz for 6 h) resulted in a moderate increase in cytoplasmic miR-204-5p levels (1.36 ± 0.07-fold vs. 0.91 ± 0.17-fold in control), the knockdown of MALAT1 using siRNA further augmented this expression significantly (2.14 ± 0.28-fold, *p* < 0.05). This observation indicates that endogenous MALAT1 putatively sequesters miR-204-5p during periods of electrical stress.

RES induced a robust upregulation of LC3B mRNA (2.47 ± 0.35-fold), comparable to rapamycin (2.56 ± 0.25-fold) (Figure 3B). Transfection with miR-204-5p wild-type mimics significantly attenuated the RES-induced increase in LC3B (1.47 ± 0.26-fold), whereas the mutant mimic had no significant effect (2.31 ± 0.14-fold). Conversely, inhibition of miR-204-5p with an antagomir further enhanced LC3B expression (2.89 ± 0.46-fold). Crucially, MALAT1 siRNA treatment effectively reversed the RES-induced upregulation of LC3B (1.27 ± 0.12-fold), mimicking the effect of 3-MA (1.26 ± 0.25-fold).

### 3.4. miR-204-5p Directly Binds to MALAT1 and the LC3B 3′-UTR

**Figure 4:**
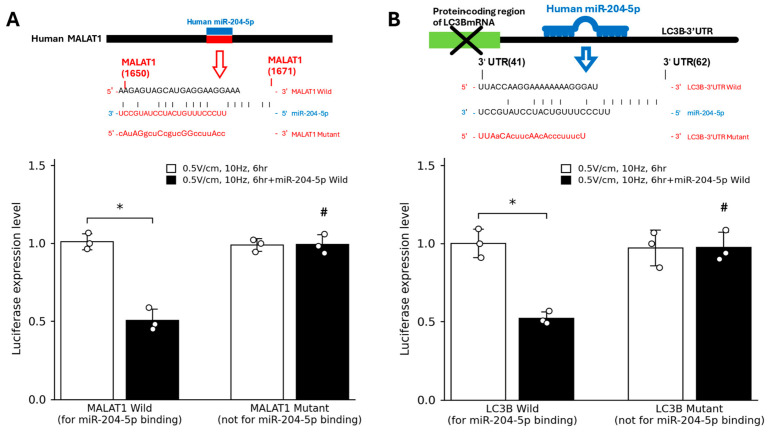
miR-204-5p directly targets MALAT1 and the 3′-UTR of LC3B mRNA. (**A**) Luciferase activity of wild-type (WT) and mutant MALAT1 constructs in HCF-aa. The schematic illustrates the predicted binding site of miR-204-5p on human MALAT1 (nucleotides 1650–1671). (**B**) Luciferase activity of WT and mutant LC3B 3′-UTR constructs. The predicted miR-204-5p binding site within the 3′-UTR of LC3B is at nucleotides 41–62. Luciferase activity was measured 24 h post-transfection and normalized to control levels. The open circles are the individual data points. Data represent mean ± SD (*n* = 3). * *p* < 0.05 vs. RES group; # *p* < 0.05 vs. RES + miR-204-5p wild-type group.

Sequence analysis revealed a predicted binding site for miR-204-5p on the MALAT1 transcript at nucleotides 1650–1671 (Figure 4A). Co-transfection with miR-204-5p wild-type mimics significantly reduced the luciferase activity of the WT-MALAT1 construct to 0.506 ± 0.071-fold compared to the RES control group (1.011 ± 0.051-fold, *p* < 0.05). Importantly, the inhibitory effect of miR-204-5p was completely abolished when the predicted binding site on MALAT1 was mutated (0.993 ± 0.072-fold), confirming that MALAT1 acts as a direct molecular sponge for miR-204-5p.

Furthermore, the predicted miR-204-5p binding site within the 3′-UTR of LC3B was located at nucleotides 41–62 (Figure 4B). MiR-204-5p mimics significantly suppressed luciferase activity in the WT-LC3B 3′-UTR, resulting in a 48% decrease (0.521 ± 0.040-fold vs. 1.001 ± 0.091-fold in the control, *p* < 0.05). This suppression was reversed upon mutation of the binding site (0.976 ± 0.098-fold).

### 3.5. The MALAT1/miR-204-5p Axis Is Essential for RES-Induced LC3B Protein Expression

**Figure 5:**
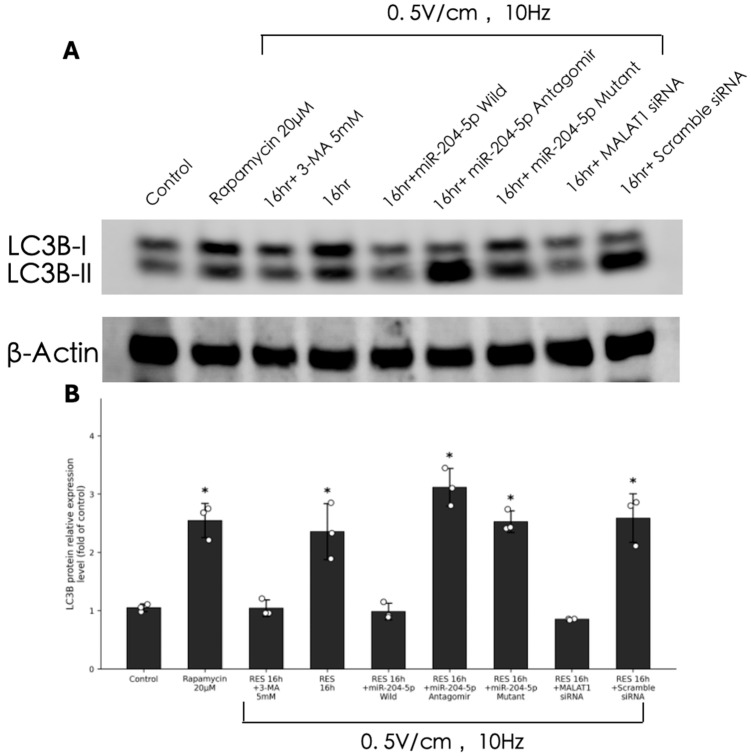
MALAT1 and miR-204-5p mediate RES-induced LC3B protein expression in HCF-aa. (**A**) Representative Western blot analysis of LC3B-I, LC3B-II, and β-actin in HCF-aa under various conditions after 16 h of RES (0.5 V/cm, 10 Hz). Conditions include control, RES alone, rapamycin (20 μM), 3-MA (5 mM), miR-204-5p wild-type/mutant mimics, miR-204-5p antagomir, and MALAT1 siRNA. Original gel images are provided in Supplementary Figure S2. (**B**) Quantitative analysis of LC3B protein levels (LC3B-II/I ratio) normalized to β-actin. The open circles are the individual data points. Data represent mean ± SD (*n* = 3). * *p* < 0.05 versus the control group.

As shown in Figure 5A, 16 h of RES significantly induced LC3B protein expression (2.36 ± 0.48-fold vs. 1.05 ± 0.06-fold in the control, *p* < 0.05), a response similar to that of rapamycin (2.55 ± 0.30-fold). The induction of LC3B was abolished by 3-MA (1.04 ± 0.15-fold), confirming activation of the autophagic pathway.

The RES-induced upregulation of LC3B protein was significantly reversed by transfection of miR-204-5p wild-type mimics (0.99 ± 0.14-fold) or MALAT1 siRNA (0.86 ± 0.01-fold), whereas miR-204-5p mutant mimics had no significant effect (2.53 ± 0.18-fold) (Figure 5B). Conversely, miR-204-5p antagomir further enhanced LC3B protein levels (3.12 ± 0.33-fold, *p* < 0.05). These protein-level findings are highly consistent with our mRNA and luciferase data, suggesting that the cytoplasmic MALAT1 pool, replenished in part through exosomal trafficking, is functionally important for LC3B-mediated autophagic activation in RES-stimulated HCF-aa; whether exosomal MALAT1 additionally acts as a paracrine mediator to recipient cells requires future conditioned media transfer experiments to confirm.

### 3.6. RES Activates Autophagy as Evidenced by LC3B Puncta Accumulation

**Figure 6:**
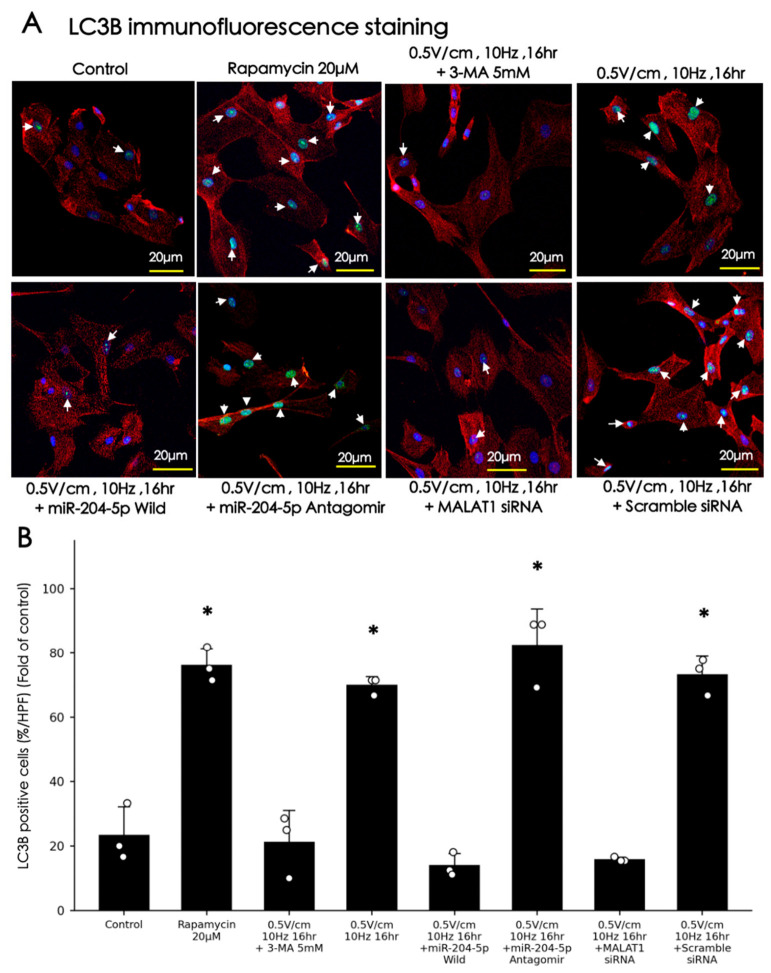
The MALAT1/miR-204-5p axis regulates autophagic activation in HCF-aa under RES, as assessed by LC3B immunofluorescence staining. Quantification reflects the percentage of LC3B-positive cells per high-power field; a validated semi-quantitative metric applied consistently across all experimental conditions. (**A**) Representative confocal microscopy images showing LC3B immunoreactive signals (green puncta, white arrows) in HCF-aa under the indicated conditions. Nuclei were counterstained with DAPI (blue), and the cytoskeleton was visualized with phalloidin (red). Scale bar = 20 μm. (**B**) Quantitative analysis of LC3B-positive cells (% per high-power field, expressed as fold of control). The open circles are the individual data points. Data represent mean ± SD (n = 3 per group). * *p* < 0.05 vs. control group.

Immunofluorescence confocal microscopy was performed to quantify LC3B-positive puncta—a well-established surrogate marker of autophagic flux [15]—in HCF-aa under various experimental conditions (Figure 6). Under baseline conditions, HCF-aa exhibited sparse LC3B signals (23.33 ± 8.82%/HPF). Rapamycin (20 μM) served as a positive control and significantly increased LC3B-positive cells (76.08 ± 5.28%/HPF, *p* < 0.05 vs. control). Consistent with Western blot data, 16 h of RES markedly increased the proportion of LC3B-positive cells (69.84 ± 2.75%/HPF, *p* < 0.05 vs. control), which was abolished by 3-MA (21.19 ± 9.85%/HPF).

Transfection with miR-204-5p wild-type mimic significantly attenuated RES-induced LC3B puncta (13.93 ± 3.75%/HPF, *p* < 0.05 vs. RES alone). Conversely, miR-204-5p antagomir further augmented LC3B accumulation (82.34 ± 11.35%/HPF). Knockdown of MALAT1 by siRNA markedly reduced RES-induced LC3B positivity (15.81 ± 0.74%/HPF), whereas scramble siRNA had no significant effect (73.15 ± 5.78%/HPF). These morphological data provide direct visual evidence that the MALAT1/miR-204-5p signaling axis is essential for RES-induced autophagic activation in atrial fibroblasts.

## 4. Discussion

This study provides novel mechanistic insights into the molecular regulation of autophagy in HCF-aa subjected to RES, focusing on the interplay among MALAT1, miR-204-5p, and the autophagy marker LC3B. While MALAT1-mediated ceRNA regulation of autophagy has been reported in other cellular contexts, including gastric cancer [16] and macrophages under hyperglycemic conditions, the present study extends this regulatory paradigm specifically to human atrial fibroblasts subjected to rapid electrical stimulation, identifying the MALAT1/miR-204-5p/LC3B axis as a potential contributor to autophagy-mediated atrial fibroblast remodeling in the context of AF.

The intracellular ceRNA mechanism is directly supported by MALAT1 siRNA rescue experiments, demonstrating that reduction of the cytoplasmic MALAT1 pool elevates miR-204-5p and suppresses LC3B; exosomal MALAT1 secretion may additionally facilitate paracrine signal propagation to neighboring cells, a mechanism validated in the same regulatory axis in our prior publication, and warrants further investigation through conditioned media transfer experiments in future studies.

RES significantly increased exosomal MALAT1 expression in a voltage- and time-dependent manner, peaking at two hours post-stimulation. Importantly, cytoplasmic MALAT1 levels did not change significantly, indicating a selective mechanism for exosomal packaging and secretion of MALAT1 in response to electrical stress [17]. MALAT1 has previously been shown to respond to diverse cellular stresses, including hypoxia and oxidative stress [8], and its rapid exosomal induction by RES suggests it may serve as an early mediator of the fibroblast stress response.

The dynamic changes in miR-204-5p expression following RES are particularly noteworthy. An initial transient upregulation at one hour was followed by a significant decline at two hours, coinciding precisely with the peak of exosomal MALAT1—an inverse temporal relationship that strongly supports the ceRNA hypothesis. The luciferase reporter assays provide direct molecular evidence, confirming that miR-204-5p binds to a specific site on the MALAT1 transcript (nucleotides 1650–1671) and that mutation of this site abolishes the inhibitory effect. MALAT1 knockdown with siRNA further corroborated the functional relevance of this sponging relationship. The growing evidence that lncRNAs modulate miRNA function through ceRNA competition [18] further supports the regulatory mechanism identified here. While luciferase reporter assays confirm direct miR-204-5p binding to MALAT1 and the LC3B 3′-UTR, they do not establish the physiological stoichiometric feasibility of ceRNA competition; the relative intracellular abundance of MALAT1 versus miR-204-5p was not quantified in this study, and future work measuring copy numbers per cell would be required to formally validate the ceRNA model under physiological conditions.

Our findings regarding LC3B expression and autophagic activation further substantiate the proposed regulatory axis. The upregulation of LC3B mRNA beginning at four hours and peaking at six hours was temporally consistent with the prior decline in miR-204-5p. The direct targeting of the LC3B 3′-UTR by miR-204-5p was confirmed by luciferase assay, establishing LC3B as a bona fide miR-204-5p target—consistent with seminal reports demonstrating miR-204-mediated suppression of LC3B in renal carcinoma [10] and in cardiomyocyte ischemia–reperfusion models [11]. Autophagic activation was further corroborated by immunofluorescence confocal microscopy, demonstrating a marked increase in cytoplasmic LC3B puncta density following RES [15]. Pharmacological modulation with rapamycin and 3-MA provided directional support for autophagic involvement; however, 3-MA exerts off-target inhibitory effects on both class I and class III PI3K and should therefore be interpreted as a pharmacological comparator rather than an autophagy-specific inhibitor. Future validation with more selective agents, such as chloroquine or ATG-targeted siRNA, would be required to confirm pathway specificity.

The clinical implications of these findings are potentially significant. The MALAT1/miR-204-5p/LC3B axis may represent a novel layer of fibroblast-specific regulation that contributes to the autophagic remodeling of the atrial substrate during AF. Fibroblast autophagy has been increasingly recognized as a determinant of fibroblast activation and myofibroblast transdifferentiation [8] processes central to atrial fibrosis and perpetuation of the arrhythmogenic substrate. Furthermore, exosomal MALAT1 may serve as a paracrine signal transmitting autophagic cues from stressed fibroblasts to neighboring cardiomyocytes [19].

Several limitations of the present study should be acknowledged. First, our experiments were conducted exclusively in vitro using HCF-aa subjected to RES, which may not fully recapitulate the complex in vivo fibrillating-atrium environment. Validation in animal models of AF and in human atrial tissue specimens will be necessary. Second, autophagic activation was assessed by LC3B expression and accumulation of puncta by immunofluorescence; direct ultrastructural confirmation by transmission electron microscopy was not performed and represents an important direction for future work. Third, the downstream consequences of LC3B-mediated autophagic activation for fibroblast function remain to be determined in future co-culture and in vivo studies. Furthermore, autophagic flux was not directly assessed using lysosomal inhibitors such as bafilomycin A1, nor was p62/SQSTM1 protein turnover measured; future studies incorporating these approaches will be necessary to definitively distinguish autophagy induction from impaired autophagic clearance. Additionally, the causal role of exosomal MALAT1 as an intercellular paracrine signal was not directly tested; experiments involving the transfer of conditioned media and the inhibition of exosome uptake would be required to formally establish its functional delivery to recipient cells. In addition, exosome isolation was performed using a validated, commercially available precipitation-based kit consistent with our previously published methodology [4]; however, comprehensive biophysical characterization, including nanoparticle tracking analysis, transmission electron microscopy, and tetraspanin marker profiling (CD63, CD81, TSG101), was not performed in accordance with MISEV2023 guidelines, and co-isolation of non-vesicular particles cannot be formally excluded. Finally, although equal protein loading was confirmed by Bio-Rad Protein Assay and LC3B values were normalized to β-actin by ImageJ densitometry, minor inter-blot variability across independent replicates is acknowledged; original unprocessed gel images are available upon request.

**Figure 7:**
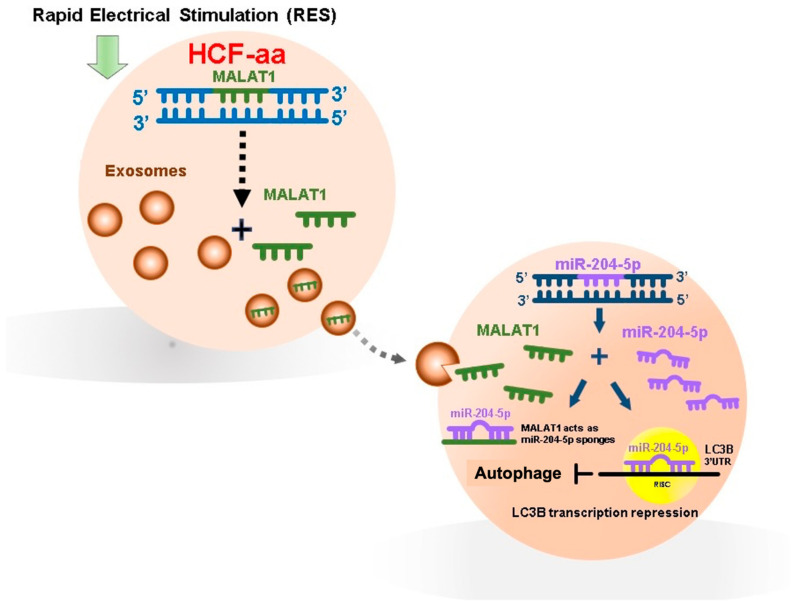
Schematic representation of the proposed molecular mechanism. Rapid electrical stimulation (RES) of HCF-aa triggers significant upregulation of exosomal MALAT1. Within the RES-stimulated HCF-aa, MALAT1 functions intracellularly as a ceRNA, potentially sequestering miR-204-5p. Concurrent exosomal secretion of MALAT1 may additionally serve as a paracrine mediator to neighboring cells, pending confirmation by conditioned media transfer experiments. This sequestration prevents miR-204-5p from binding to the 3′-UTR of LC3B mRNA, thereby relieving miR-204-5p-mediated repression of LC3B expression. The resulting upregulation of LC3B activates autophagic signaling in atrial fibroblasts subjected to rapid electrical stress.

In conclusion, our study reveals a novel regulatory axis in which RES-induced exosomal MALAT1, as a ceRNA, putatively sequesters miR-204-5p, thereby derepressing LC3B expression and activating autophagy in atrial fibroblasts (Figure 7). These findings advance our understanding of the regulation of non-coding RNA-mediated autophagy in atrial fibroblasts under electrical stress and suggest that the MALAT1/miR-204-5p/LC3B axis may serve as a potential therapeutic target for modulating autophagy-mediated atrial structural remodeling.

## Data Availability

The original contributions presented in this study are included in the article/Supplementary Materials. Further inquiries can be directed to the corresponding author.

## Supplementary Materials

The following supporting information can be downloaded at: https://www.mdpi.com/article/10.3390/cells15121126/s1, Figure S1: Original gel images used in Figure 2C (time-course Western blot of LC3B-I and LC3B-II in HCF-aa subjected to RES for 1–24 h; *n*  =  3 per group); Figure S2: Original gel images used in Figure 5A (Western blot of LC3B-I and LC3B-II in HCF-aa under various functional interventions after 16 h of RES; *n*  =  3 per group).
