# Supplementary material for: Exosomal MALAT1 from Rapid Electrical Stimulation-Treated Atrial Fibroblasts Activates Autophagy by Downregulating miR-204-5p and Upregulating LC3B
Source: Cells. 2026 Jun 22;15(12):1126. doi: 10.3390/cells15121126 (PMC13297054; doi:10.3390/cells15121126)
Supplement: Supplementary file 1 [file cells-15-01126-s001.zip › cells-4383505-supplementary.pdf]

Exosomal MALAT1 from Rapid Electrical Stimulation-Treated Atrial Fibroblasts Activates Autophagy by Downregulating miR-204-5p and Upregulating LC3B

Su-Kiat Chua[1,2,*], Bao-Wei Wang[2], Ying-Ju Yu[2], Wei-Jen Fang[2], Chiu-Mei Lin[1,3], Cheng-Yen Chuang[2] and Kou-Gi Shyu[2]

1 School of Medicine, College of Medicine, Fu Jen Catholic University, New Taipei 24205, Taiwan

2 Division of Cardiology, Department of Internal Medicine, Shin Kong Wu Ho-Su Memorial Hospital, Taipei 11101, Taiwan

3 Department of Emergency Medicine, Shin Kong Wu Ho-Su Memorial Hospital, Taipei 11101, Taiwan

* Correspondence: benchua1131@hotmail.com

Corresponding Author:

Su-Kiat Chua, MD, PhD

Division of Cardiology

Department of Internal Medicine

Shin Kong Wu Ho-Su Memorial Hospital

No. 95, Wen Chang Road, Shih-Lin District, Taipei, Taiwan

Tel: 886-2-2833-2211 ext. 2084

Fax: 886-2-2836-5775

E-mail: benchua1131@hotmail.com

Supplementary Materials

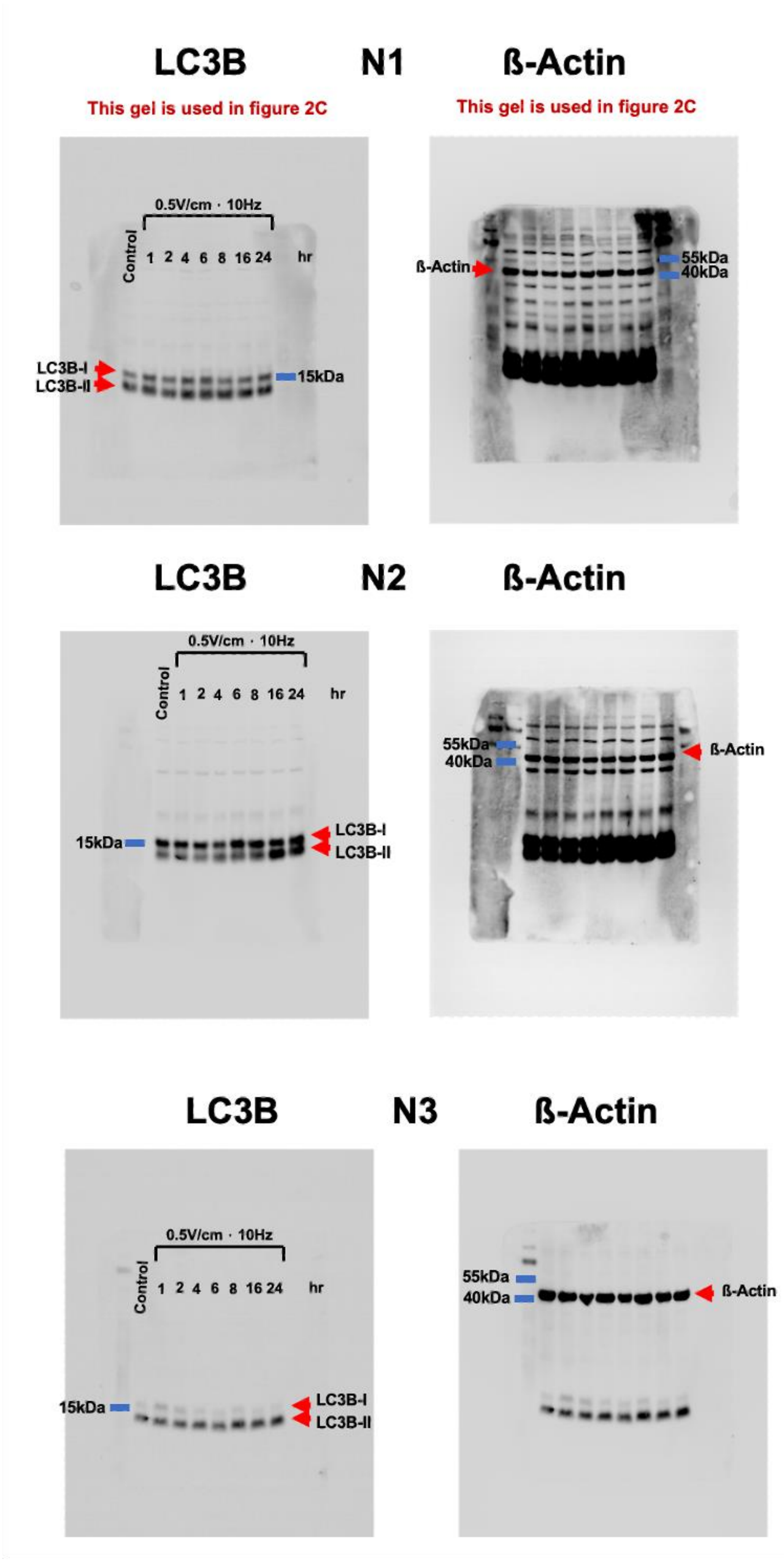

Supplementary Figure S1: Original gel images used in Figure 2C (time-course Western blot of LC3B-I and LC3B-II in HCF-aa subjected to RES for 1–24 hours; n = 3 per group).

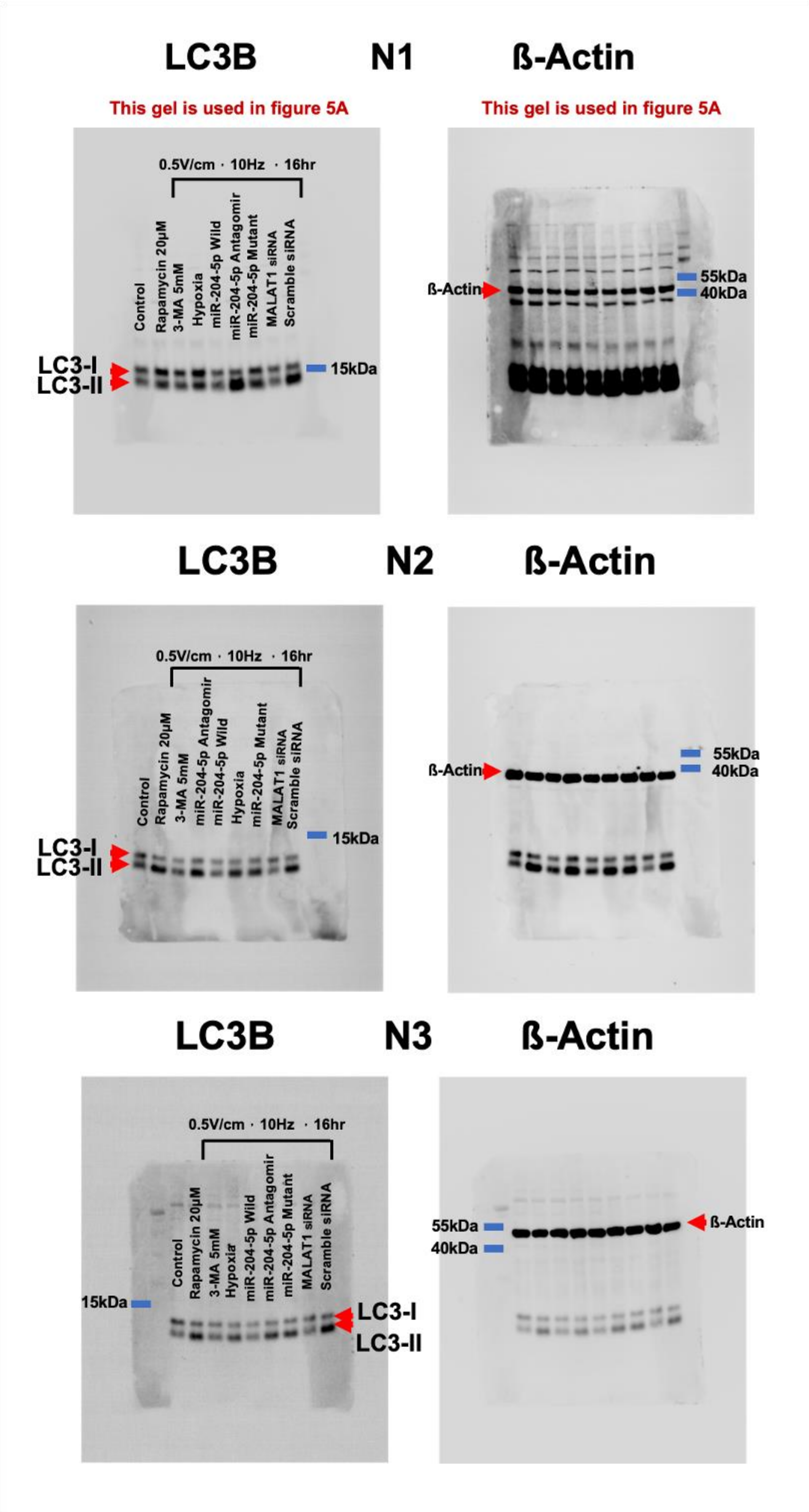

Supplementary Figure S2: Original gel images used in Figure 5A (Western blot of LC3B-I and LC3B-II in HCF-aa under various functional interventions after 16 hours of RES; n = 3 per group).
